# Amelioration of Cytogenotoxic Damage in Drug Abusers Supplemented with Folic Acid

**DOI:** 10.3390/biomedicines12020352

**Published:** 2024-02-02

**Authors:** Alejandro Salvador Gómez-Cabrera, Ana Elizabeth González-Santiago, José Francisco Rodríguez-Mora, Guillermo Moisés Zúñiga-González, Belinda Claudia Gómez-Meda, Raúl Cuauhtémoc Baptista-Rosas, Rolando Castañeda-Arellano, Arieh Roldán Mercado-Sesma, Laura Yareni Zúñiga, María Guadalupe Sánchez-Parada

**Affiliations:** 1 Departamento de Ciencias Biomédicas, División de Ciencias de la Salud, Centro Universitario de Tonalá, Universidad de Guadalajara, Tonalá 45425, Jalisco, Mexico; alejandro.gomez9676@alumnos.udg.mx (A.S.G.-C.); jose.rodriguez7316@alumnos.udg.mx (J.F.R.-M.); 2 Laboratorio de Mutagénesis, Centro de Investigación Biomédica de Occidente, Instituto Mexicano del Seguro Social, Guadalajara 44340, Jalisco, Mexico; mutagenesis95@hotmail.com; 3 Instituto de Genética Humana Dr. Enrique Corona Rivera, Departamento de Biología Molecular y Genómica, Centro Universitario de Ciencias de la Salud, Universidad de Guadalajara, Guadalajara 44340, Jalisco, Mexico; belinda.gomez@academicos.udg.mx; 4 Departamento de Ciencias de la Salud-Enfermedad como Proceso Individual, Centro Universitario de Tonalá, Universidad de Guadalajara, Tonalá 45425, Jalisco, Mexicorolando.castaneda@academicos.udg.mx (R.C.-A.); lauray.zuniga@academicos.udg.mx (L.Y.Z.); 5 Unidad de Intervención de Medicina Crítica, Hospital General de Occidente, Secretaría de Salud Jalisco, Zapopan 45170, Jalisco, Mexico

**Keywords:** cytogenotoxicity, folic acid, buccal micronucleus cytome assay, addicts

## Abstract

Background: Cytogenotoxic damage caused by the consumption of legal and illegal drugs in drug abusers has been demonstrated, primarily due to alterations in their antioxidant capacity, cellular repair mechanisms, and increased production of free radicals. Folic acid shows antioxidant activity by acting as a reducing agent, neutralizing present free radicals, and reducing genomic damage. Methods: The intervention involved administering 15 mg of folic acid, divided into three doses per day, to a group of 44 drug abusers. The frequency of nuclear abnormalities (NAs) was determined; micronuclei (MNs), nuclear buds (NBUDs), binucleated cells (BNs), abnormally condensed chromatin (CC), karyorrhexis (KX), pyknotic nuclei (PNs), and karyolysis (KL) were determined at different pre-treatment (baseline) and post-treatment time points at 15 and 30 days. Additionally, a group of 44 healthy individuals was used as the control group. Results: We observed a statistically significant decrease in the frequency of NAs in the drug abuser group (28.45 ± 17.74 before supplementation vs. 11.18 ± 7.42 at 15 days and 9.11 ± 10.9 at 30 days of supplementation). Specifically, it decreased the frequency of NBUDs, BNs, CC, KX, and PNs (*p* < 0.05). Conclusion: Our study demonstrates a clear improvement in cytogenotoxic damage in drug abusers supplemented with folic acid.

## 1. Introduction

The growing global drug abuse has turned into a significantly widespread public health issue, coupled with socio-economic implications [1,2,3,4,5,6].

Multiple studies have demonstrated the deleterious effect of legal and illegal drug consumption on the integrity of genetic material and cellular structure. This cytogenotoxic effect has been associated with alterations in DNA repair enzymes, limitations in antioxidant capacity, an increase in the production of free radicals, inflammation, and alterations in cellular repair and proliferation [7,8,9,10,11,12,13,14].

Folic acid (vitamin B9) is necessary for various metabolic processes, as it is essential for the synthesis of nucleic acids and required for the proper growth of fetuses. In addition, its deficiency can lead to the onset of megaloblastic anemia and neural tube defects in fetuses, as well as the development of cardiac diseases and certain cancers [15].

The metabolism of folates is involved in three intracellular processes, where they are used as coenzymes for the synthesis of thymidylate and purines, which are precursors for the synthesis of deoxyribonucleic acid (DNA) and ribonucleic acid (RNA). It also includes the synthesis of methionine from homocysteine and the interconversion of serine and glycine [16]. Additionally, it shows antioxidant activity by acting as a reducing agent, neutralizing present free radicals, and restoring the bioavailability of nitric oxide by interacting with nitric oxide synthase and increasing the availability of the cofactor tetrahydrobiopterin [17]. There is scientific evidence demonstrating the antioxidant effect of folic acid in improving genetic damage through its protective effect, as evidenced by the reduction in the frequency of nuclear abnormalities [18].

The buccal micronucleus cytome (BMCyt) assay is an accessible method for the detection of cytogenotoxic damage. It is used to detect cellular and genetic material damage induced by a substance of interest [19,20].

Additionally, the BMCyt assay is employed as a predictive marker, as micronuclei and other nuclear abnormalities (NA) serve as indicators of DNA damage and cell death. Their increase is associated with genotoxic, cytotoxic, and cancerous processes [21,22,23,24,25].

NAs include micronuclei (MNs), seen as fragments of chromosomes or complete chromosomes, surrounded by a nuclear membrane, which appear as small nuclei in the interface, as they do not incorporate into the daughter cells during mitosis. This occurs due to the effects of clastogenic or aneuploidogenic agents. Nuclear buds (NBUDs) represent the expression of amplified genes under specific conditions. These genes are positioned at the periphery of the nucleus and, subsequently, extruded from the nucleus in the form of a bud. Binucleated cells (BNs) are cells with two main nuclei. The significance of their formation is still unknown, but it is associated with a failure in cytokinesis and a defect in the mitotic spindle. Cells with abnormally condensed chromatin (CC) exhibit a fragmented nucleus (striated pattern), which will ultimately lead to nuclear disintegration, representing an early stage of the apoptotic process. Cells with karyorrhexis (KX) display a nucleus with extensive nuclear fragmentation (mottled pattern). It represents an advanced stage of the apoptotic process, which will also culminate in nuclear disintegration. Pyknotic nuclei (PNs) are reduced-size and very dense nuclei, representing an alternative form of nuclear disintegration. Cells exhibiting karyolysis (KL) are cells without a nucleus, representing an advanced state of cell death [19,20,26,27].

In this study, we evaluated the effect of folic acid supplementation on nuclear abnormalities induced by cytogenotoxic damage in addicts through the BMCyt assay. We analyzed baseline samples (pre-treatment) and samples at 15 and 30 days (post-treatment).

## 2. Materials and Methods

### 2.1. Study Population

The present project was conducted on individuals with a history of legal and illegal drug abuse, institutionalized at the “Quinta Sobriedad Emocional A.C.” rehabilitation center in the municipality of El Salto, Jalisco, México. A total of 44 participants of both genders were included in this study after obtaining informed consent. Additionally, a control group consisting of 44 healthy individuals with no history of drug consumption from the metropolitan area of Guadalajara, Jalisco, Mexico, was included.

### 2.2. Questionnaire and Oral Examination

A confidential questionnaire was realized prior to sampling to gather general information, medical and non-medical history, as well as detailed information on the type of drugs used, their quantity, and duration of consumption. This allowed for the selection of participants who met the inclusion criteria. The questionnaire included age, gender, occupation, personal medical history, medications, family history, eating habits, exposure to known carcinogens, and radiation. Finally, patterns of alcohol, tobacco, marijuana, methamphetamine, cocaine, and inhalant consumption were identified. After completing the questionnaire, an examination of the oral cavity was conducted to identify any lesions. This evaluation was carried out by a professional dentist.

All participants met the following criteria: men or women aged 18 years or older and without concomitant diseases (type 1 and type 2 diabetes mellitus, systemic arterial hypertension, chronic kidney disease, periodontal disease, cancer). They were also individuals without prior consumption (three months before) of any supplements or vitamins such as folates, B12, C, E, A, lutein, or melatonin, or any other antioxidant, medications for the pharmacological treatment of chronic diseases, or that interfere with folic acid metabolism (acetazolamide, amiloride, benzthiazide, bromothiofiline, bumetanide, canrenoic acid, chlorothiazide, chlortalidone, colestipol, cyclopenthiazide, cyclothiazide, cyclofenamide, drospirenone, and other diuretics). Other requirements included individuals without active, severe periodontal disease, without exposure to fertilizers, radiation, or heavy metals, or individuals living in areas with high levels of air pollution.

Individuals who consumed legal (alcohol, tobacco) and illegal (marijuana, cocaine, methamphetamines, inhalants) drugs during the last 6 months, at least one of them in the following doses, were included: alcohol (more than 4 standard drink units (SDU) consumed per day), tobacco expressed as smoking index (cigarrettes smoked per day per years of consumption) greater than 4, marijuana (more than 18 g per week), cocaine (more than 1 g per week), methamphetamines (more than 7 g per week), and inhalants (more than 6 h per week of inhalation). Pregnant or breastfeeding individuals were excluded.

### 2.3. Supplementation with Acid Folic

An oral supplementation was carried out using 5 mg of folic acid tablets of the brand Ciclocid (Bruluagsa, Atlacomulco de Fabela, Estado de Mexico, México) every 8 h over the course of one month. The supplement was administered by the nursing staff at the rehabilitation center “Quinta Sobriedad Emocional A.C.” in El Salto, Jalisco, Mexico. They meticulously monitored the supplementation through a consumption diary and conducted a brief questionnaire to monitor any potential adverse effects (no adverse effects were reported during this study).

### 2.4. Collection, Preparation, and Analysis of Samples Using the BMCyt Assay

Samples of oral mucosa cells were collected from each participant by direct scraping with a beveled glass slide on the cheek at baseline on day 0 (pre-treatment) and at 15 and 30 days (post-treatment). The sample was evenly distributed on duplicate-coded glass slides, allowed to air dry, and stored for subsequent fixation in absolute methanol for 48 h. The samples were stained with acridine orange (Sigma-Aldrich, Burlington, MA, USA), a nucleic acid-specific dye, before the analysis of nuclear abnormalities.

Nuclear abnormalities, including MNs, NBUDs, BNs, CC, KL, KX, and PNs, were analyzed. A total of 2000 cells/sample were examined using a fluorescence microscope (Olympus BX51, Tokyo, Japan) with a 100-fold× immersion objective. Two observers (blinded to the study groups and not involved in sample coding) assessed the presence of nuclear abnormalities in all collected samples according to established guidelines.

### 2.5. Statistical Analysis

The data analysis was conducted using GraphPad 9.2.0 for Windows. For continuous quantitative variables, the data were presented as measures of central tendency and dispersion: mean and standard deviation. For ordinal quantitative variables, frequencies and percentages were presented. The statistical analysis to find differences between groups (healthy control vs. baseline drug consumers) was performed using the Mann–Whitney U test. The statistical analysis to find differences between baseline (pre-treatment) drug consumers and 15 and 30 days (post-treatment) was performed using ANOVA (Dunnett’s multiple comparisons).

## 3. Results

### 3.1. Demographic Characteristics and Drug Consumption Pattern

The general characteristics of all participants in terms of gender and age are displayed in groups. The drug abuser group comprised a sample of 44 individuals with a history of legal and illegal drug consumption. The drug’s abuser group included the following: 7 individuals consuming tobacco, alcohol, cocaine, and methamphetamine; 8 individuals consuming tobacco, alcohol, marijuana, and methamphetamine; 9 individuals consuming tobacco, alcohol, marijuana, cocaine, and methamphetamine; 9 individuals consuming tobacco, alcohol, marijuana, methamphetamine, and inhalants; and 11 individuals consuming tobacco, alcohol, marijuana, cocaine, methamphetamine, and inhalants. Additionally, a group of 44 healthy individuals in the control group were included. The gender distribution showed a predominance of males in the group of drug abusers, with 37 male participants and 7 females, with a mean age of 29.54 ± 9.38. The control group comprised 17 males and 27 females, with a mean age of 31.16 ± 9.36 (Table 1).

Additionally, we analyzed the consumption patterns of the group with drug abuse. The mean smoking index was 5.92 ± 6.61, with a mean of 4.85 ± 6.95 SDU per day. The weekly consumption of marijuana showed a mean of 18.29 ± 27.65 g, while cocaine use exhibited a mean of 1.32 ± 2.36 g per week. The weekly methamphetamine intake disclosed a mean of 7.48 ± 9.59 g. Furthermore, the average number of hours dedicated to inhalant consumption per week was 6.15 ± 14.76 (Table 1).

### 3.2. Frequency of Nuclear Abnormalities in the Drug Abuser Group vs. the Control Group

**Figure 1:**
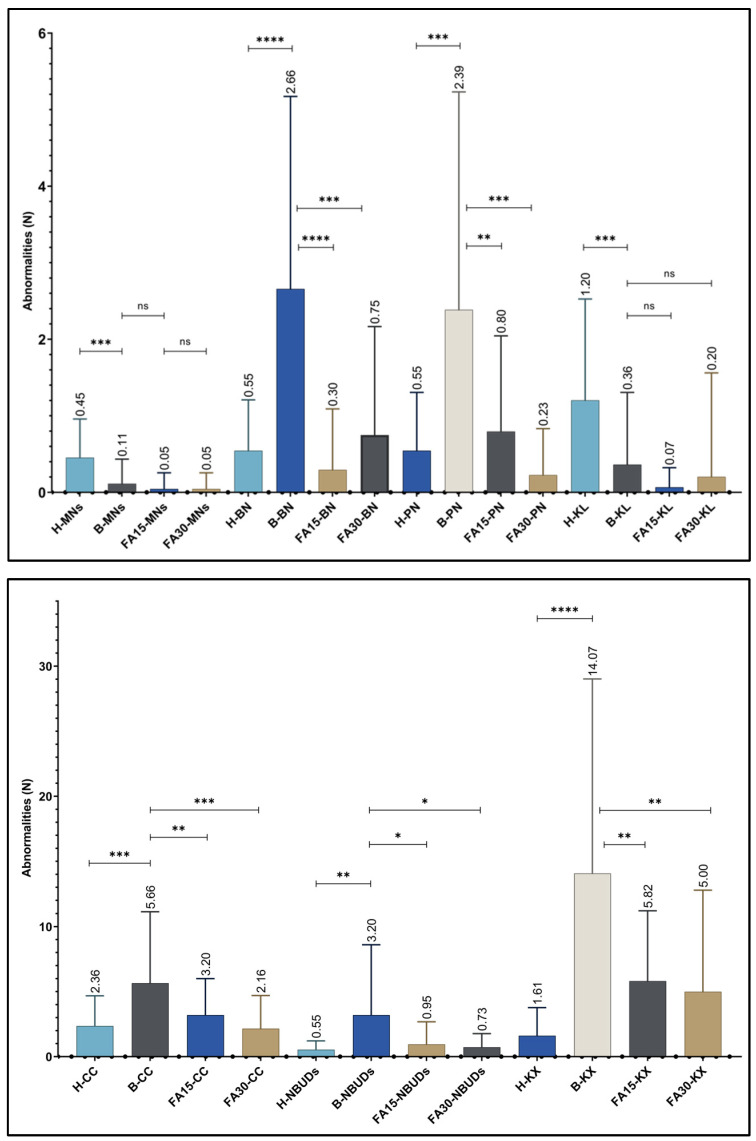
Comparison of the frequency of nuclear anomalies between the healthy control group, baseline drug abusers (pre-treatment), and 15 and 30 days (post-treatment) with folic acid. Control group healthy (H), baseline pre-treatment (B), and 15- and 30-days post-treatment with folic acid (FA 15 and FA 30). Micronuclei (MNs), nuclear buds (NBUDs), binucleated cells (BNs), abnormally condensed chromatin (CC), karyorrhexis (KX), karyolysis (KL), and pyknotic nuclei (PNs). Statistical significance was considered with a value of *p* < 0.05 (*), *p* < 0.005 and < 0.001 (**), *p* < 0.0005 (***) and <0.0001 (****), and no statistical significance (ns).

**Figure 2:**
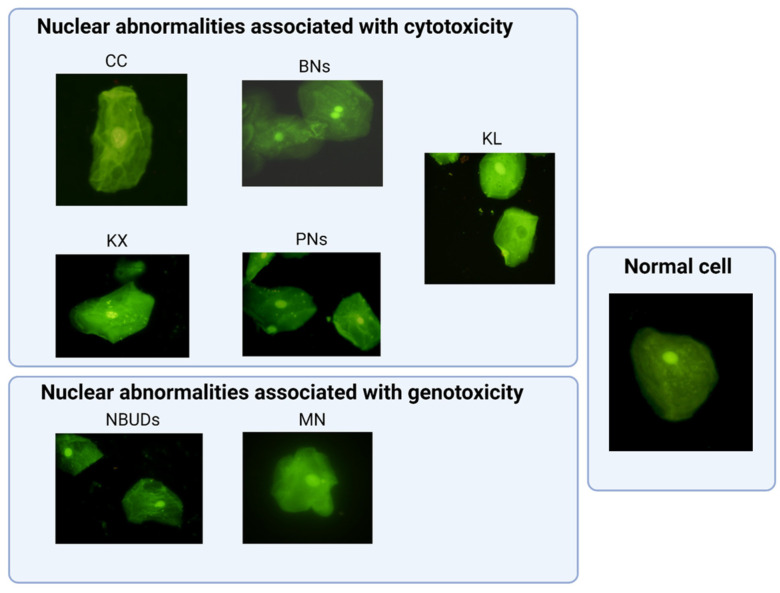
Representative buccal epithelial cells with different types of nuclear abnormalities and a normal cell. CC (condensed chromatin), BNs (binucleated cells), KX (kariorrhectic cell), PNs (pyknotic cells), KL (karyolytic cell), NBUDs (nuclear buds), and MN (micronucleus).

The frequency of nuclear abnormalities through the BMCyt assay was evaluated in the drug abuser group and the healthy control group. The nuclear abnormalities found in each group were compared, revealing statistically significant differences between both groups, as shown in Table 2 and Figure 1 and Figure 2. The frequency of total nuclear abnormalities was higher in the drug abuser group compared to the control group, with a mean of 28.45 ± 17.74 and 9.09 ± 3.79, respectively (*p* < 0.001). The micronuclei (MNs) were found to be at a lower frequency in the drug abuser group compared to the control group (*p* < 0.0001). There was an increase in the frequency of nuclear buds (NBUDs) in the drug abuser group compared to the control group (*p* < 0.005). An increase in the frequency of binucleated cells (BNs), nearly five times higher, was observed in the drug abuser group compared to the control group (*p* < 0.0001). The abnormally condensed chromatin (CC) had a frequency more than two-fold higher in the group of interest compared to the healthy controls (*p* < 0.0005). Karyorrhexis (KX) exhibited a marked increase in the addict’s group, with values found to be eight times higher compared to the control group (*p* < 0.0001). KX was identified as the nuclear abnormality most frequently found in this study. When analyzing the frequencies of pyknotic nuclei (PNs) in the drug abuser group, values were observed to be more than four times higher than those found in the healthy group (*p* < 0.0005) (Table 2).

Depicted in Figure 1 is the marked difference in the frequency of nuclear abnormalities between the healthy control group and the drug abuser group in the bars identified as (H) and (B).

### 3.3. Frequency of Nuclear Abnormalities in the Drug Abuser Group vs. the Folic Acid Treatment Group

A comparative analysis was conducted between the frequency of nuclear abnormalities found in baseline (pre-treatment) drug consumers and the frequencies found at 15 and 30 days (post-treatment) with folic acid. A statistically significant decrease was demonstrated in the majority of nuclear abnormalities, as described below:

There was a decrease in the frequency of nuclear buds (NBUDs) more than three times at 15 days post-treatment and more than four times at 30 days post-treatment (*p* < 0.001). The binucleated cells (BNs) showed a marked decrease in their frequency of over eight times at 15 days post-treatment and over three times at 30 days post-treatment (*p* < 0.0001) (*p* < 0.0005), respectively. There was a decrease in the frequency of abnormally condensed chromatin (CC) evaluated at 15 days post-treatment, and at 30 days post-treatment, there was a decrease of more than double of this nuclear abnormality (*p* < 0.005) (*p* < 0.0005), respectively. Karyorrhexis (KR) decreased its frequency by more than double at 15 days post-treatment and almost three times at 30 days post-treatment (*p* < 0.0005). Interestingly, a decrease in the frequency of pyknotic nuclei (PNs) was found to be almost three times at 15 days post-treatment, but the decrease at 30 days post-treatment was more than ten times compared to baseline measurement (*p* < 0.005) (*p* < 0.0001), respectively. Although not statistically significant, a decrease in the frequency of micronuclei (MNs) was also observed at 15 and 30 days post-treatment, as well as karyolysis (KL) at 30 days post-treatment (Table 3).

In Figure 1, the schematic representation illustrates the clear decrease in nuclear abnormalities upon receiving supplementation. This is depicted in the bars identified as baseline pre-treatment (B) and 15- and 30-days post-treatment with folic acid (FA 15 and FA 30).

## 4. Discussion

### 4.1. Effect of Drug Abuse on the Occurrence of Nuclear Abnormalities

In this study, we assessed the cytogenotoxicity using the BMCyt assay in a group of healthy individuals and a group with drug abuse to determine the effect of drugs on genetic material and cellular structure. We analyzed nuclear anomalies associated with genotoxicity (MNs and NBUDs), stages of early (CC) and late (KR) apoptotic processes, alternative forms of nuclear disintegration (PNs), cytokinesis defects (BNs), and advanced stages of cell death (KL). Multidrug use is more frequent in the drug-using population than single-drug use. However, most studies are focused on the evaluation of individual drugs, which has certainly contributed to the knowledge of their cytogenotoxic damage. The higher frequency of NAs and the results of this study are consistent with those reported by other authors [10,24,28,29,30,31,32,33,34,35,36], since in this study, a statistically significant difference in the frequency of (NAs) was found between subjects with drug abuse (28.45 ± 17.74) compared to non-drug users (healthy controls (9.09 ± 3.79)).

In a study conducted by de Geus JL et al., 16 cross-sectional clinical trials were selected, comparing the frequency of MNs in the oral mucosa of adult smokers and non-smokers. The results show a higher frequency of MNs in the exfoliated cells of smokers compared to non-smokers [37]. Similarly, in a study conducted by Abdul NS et al., 20 studies were analyzed in which cytogenetic abnormalities were assessed in the oral mucosa of waterpipe tobacco smokers (WTS) and cigarette smokers, finding higher frequencies of MNs in WTS compared to non-smokers [38].

Moreover, in a study conducted by Nersesyan A et al., MNs and other nuclear abnormalities were analyzed to investigate the impact of nicotine and tar from different types of cigarettes. The frequencies of KR, CC, KL, NBUDs, and BNs increased significantly only in medium-filter (MF) and non-filtered (NF) cigarette smokers, while MNs elevated only in NF smokers. They suggest that some nuclear abnormalities are more sensitive assessment criteria than MNs, given that these abnormalities only increased with lower exposure to the toxic components of tobacco. An increase in MN frequency was only observed with higher amounts of nicotine and tar in consumed cigarettes [39].

Furthermore, a study conducted by Naderi NJ et al. determined the frequency of MNs in a group of individuals with a history of smoking for less than 10 years, as well as in a group with a history of more than 10 years of tobacco consumption. They found a statistically significant difference in the frequency of MNs in the buccal mucosa cells of non-smokers compared to smokers. The frequency of MNs in the group of smokers with more than 10 years of tobacco consumption was higher than that found in the group of smokers with less than 10 years of tobacco consumption [40].

The results of previous studies support the relationship between tobacco consumption and the increase in MNs and other NAs. However, it should be taken into consideration that the frequency of these abnormalities may be modified by the type of cigarettes consumed as well as by the years of tobacco consumption [37,38,39,40,41,42].

Likewise, tobacco use has been associated with a cascading effect on the production of free radicals and oxidants [43]. Moreover, exposure to cigarette smoke has been linked to markers of mitochondrial stress, inflammation, and senescence [44], providing a clear insight into why this drug causes damage.

In line with our results evaluating alcohol effects, in a study conducted by Rocha R et al., they evaluated the presence of MNs and other NAs (KX, PNs, CC) in three exposed groups (mouthwash users, alcohol consumers, and mouthwash and alcohol users), as well as a group not exposed to alcohol. It was found that mouthwashes alone or combined with alcoholic beverages induce chromosomal damage and apoptosis. Apoptosis was evaluated through the sum of parameters KX, PNs, and CC, which was significantly higher in the exposed groups. Additionally, it was observed that alcoholic beverages can independently stimulate the apoptosis process [45].

Similarly, the results reported in a study by Zamora-Perez AL et al. demonstrated that the use of mouthwash with alcohol significantly increases the frequency of MNs and other nuclear abnormalities (NBUDs, KL, KR, CC) in exposed individuals compared to users of alcohol-free mouthwash [46].

In this same line, in a study conducted by Reis SR et al., they analyzed the presence of MNs in exfoliated cells from the tongue and oral mucosa in two groups (alcoholics with no history of smoking and abstainers from both alcohol and tobacco). They found a significant increase in the frequency of MNs in the exfoliated cells from the tongue in the alcoholic group compared to the abstinent group. Likewise, they also found an increase in the frequency of MNs in cells from the oral mucosa, although this increase was not significant [47].

Additionally, the metabolic pathways through which this drug causes oxidative stress, DNA damage, and the formation of adducts, as well as mitochondrial dysfunction, have been elucidated [48,49,50,51,52,53,54].

It has been demonstrated that smoked marijuana, either alone or in combination with tobacco, is capable of inducing genetic damage and cytotoxicity in the cells of the oral mucosa and peripheral blood of consumers. This is evidenced by a study conducted by Souza DV et al., where there was an increase in MNs and KL, as well as by a study carried out by Fabian-Morales E et al., in which genotoxic damage was also demonstrated through an increase in MNs and BUDs [24,55].

Furthermore, the cellular and molecular mechanisms by which cannabinoids can cause damage are known. Their deleterious effects include an increase in reactive oxygen species, uncoupling of oxidative phosphorylation, activation of oncogenic pathways, inhibition of DNA repair enzymes, and alterations in telomere repair [56,57,58,59].

In line with our results, a study conducted by Almeida TC et al., which evaluated buccal mucosa cells of cocaine users, showed a statistically significant increase not only in MNs and PNs but also in the abnormality KX [60]. KX nuclear change was the most frequent in the exposure group, consistent with the findings of our present study. In other in vitro and in vivo studies, oxidative stress, genotoxic damage, inflammation, cell death, and mutagenicity induced by cocaine have been detected [8,23,61].

In studies conducted on cell cultures exposed to methamphetamines, the induction of MNs and NBUDs was observed, resulting from chromosomal aberrations and genetic amplifications. Additional experiments confirmed the oxidation of purines and pyrimidines, indicating that genotoxic damage occurs through DNA oxidation pathways [62]. In accordance with this, a study conducted by Li JH et al. reports the genotoxic effects induced by methamphetamines, highlighting the crucial role played by reactive oxygen species in causing the damage [63].

In a study conducted by Martínez-Alfaro M et al., their analysis of animal models exposed to thinner did not show a specific genotoxic effect; however, an induction of oxidative stress was demonstrated [64]. Likewise, benzene (found in gasoline) has shown a direct effect on hematopoiesis, with the ability to generate acute myeloid leukemia [65].

The components of thinners (toluene, benzene, acetone, ethanol, butanol, and xylenes) induce oxidative stress as well [64].

The presented studies support our results regarding a higher frequency of nuclear abnormalities in the drug abuser group.

### 4.2. Effect of Folic Acid Supplementation on the Occurrence of Nuclear Abnormalities in Drug Abusers

In our study, we assessed the effect of folic acid supplementation on individuals with drug abuse to determine its beneficial impact on the amelioration of nuclear abnormalities. This was based on the premise that several studies have confirmed its protective effects and antioxidant capacity [66,67,68,69].

We analyzed genotoxicity abnormalities (MNs, NBUDs) and cytotoxicity (CC, KX, PNs, BNs, KL) both at baseline (pre-treatment) and subsequently at 15 and 30 days (post-treatment) with folic acid. When comparing baseline measurements with post-treatment measurements, we confirmed a decrease in all evaluated parameters, with a statistically significant decrease in NBUDs, CC, KX, PNs, and BNs.

Multiple studies have reported on the significant role of folic acid in preventing genetic damage and reducing mutagenicity [70,71,72,73,74,75,76,77,78]. Hence, we could attribute such capability to the improvement observed in our supplemented group.

In this regard, a study conducted by Matté C et al. successfully demonstrated that the deleterious effects on DNA caused by the chronic application of homocysteine in an animal model were avoided with the simultaneous application of folic acid, suggesting that its possible antioxidant capacity and DNA stability were responsible for such an effect [70].

As a coadjuvant in DNA stability and damage reduction, Stopper H et al. demonstrated in hemodialysis patients that through supplementation with folic acid and vitamin B12, they achieved a decrease in genomic damage to peripheral blood lymphocytes, along with a trend in the reduction of 1,N^6^-etheno-2′deoxyadenosine, a marker of oxidative stress [74].

In a study conducted by Zhang R et al., the effect of folic acid on the mutagenicity and genotoxicity caused by benzo(a)pyrene was investigated through in vitro and in vivo experiments. Their results demonstrated a protective effect of folic acid on the viability of human liver cells. Similarly, a decrease in the number of MNs was found in the supplemented groups [76].

Emphasizing the impact of folate concentration on DNA integrity is important, as our study group is affected not only by multidrug consumption but also by dietary deficiencies. Fenech et al. mention that folic acid plays a key role in preventing chromosomal and DNA damage [79]. It also points out a possible explanation for chromosomal damage at low folate levels since erroneous and excessive incorporation of uracil into DNA may occur, which could lead to chromosomal breaks, fragile sites, the formation of micronuclei, and DNA hypomethylation. In vitro experiments indicate that genomic instability is minimized at concentrations of >227 nmol/L of folic acid. In humans, the concentration in red blood cells is >700 nmol/L of folate, and micronuclei formation is minimized at plasma concentrations of >300 pmol/L of vitamin B12 and <7.5 micromole/L of homocysteine. These concentrations are achievable with an intake of more than 200 to 400 micrograms of folic acid per day and more than 2 micrograms of vitamin B12 per day. Dietary intake plays an important role. Under this premise, there is a correlation between vitamin B12 deficiency and high levels of homocysteine and the formation of micronuclei, as well as a decrease in genomic instability after supplementation with folic acid [79].

Similarly, in a study conducted by Beetstra S et al., they demonstrated that folic acid deficiency can increase genomic damage induced by radiation. This can be explained because folic acid deficiency can lead to hypomethylation and deficient DNA repair, as well as chromosomal breakage, increasing the sensitivity of ionizing radiation to cause genetic instability [77].

Abramsson-Zetterberg L et al. conducted two intervention studies in which they provided different nutritional supplements to individuals without folate deficiency and observed if these had any effect on the frequency of MNs in human transferrin-positive reticulocytes, as well as on cell proliferation (percentage of polychromatic erythrocytes). In the first study, participants were supplemented with 1 mg/day of folic acid for 1 week. In the second study, supplementation included 800 micrograms of folic acid per day, 20 micrograms of vitamin B12 per day, and 4 micrograms of vitamin B6 per day. No significant differences were found between the intervention groups [80].

In our study, we conducted supplementation with 15 mg of folic acid daily, divided into three doses, for one month. We considered that the amount and frequency of folic acid administration in participants are related to the results obtained, which are consistent with those reported by Gómez-Meda et al., in which supplementation with folic acid was evaluated in patients with type 1 and type 2 diabetes mellitus. This supplementation was also carried out with 5 mg of folic acid tablets in a total of three doses over one month. They also found a decrease in nuclear abnormalities (NAs) before and after folic acid supplementation (20.4 ± 8.0 before treatment vs. 10.5 ± 5.2 after treatment). They attribute the statistically significant decrease in NAs (MNs, BNs, PNs, KX, KX+CC, KL, NBUDs) to folic acid supplementation [18].

The results found in our study align with [18], as we also observed a statistically significant decrease in the frequency of NAs in the drug abuser group (28.45 ± 17.74 before folic acid supplementation vs. 11.18 ± 7.42 at 15 days of supplementation and 9.11 ± 10.9 at 30 days of supplementation), with a decrease in the frequency of all evaluated nuclear abnormalities, with statistical significance in NBUDs, CC, KX, PNs, and BNs.

Based on the premise of its clear antioxidant capacity and its effect on genomic integrity, we attribute the amelioration of cytogenotoxic damage in drug abuser patients to supplementation with folic acid at a dose of 15 mg per day for one month. As with other schemes, no improvement was observed in the evaluated parameters. However, we are aware of the need for further studies to find the initial and maintenance dose to sustain such improvement, that is, studies that allow us to assess the minimum therapeutic dose of folic acid in improving nuclear abnormalities in drug abusers.

## 5. Conclusions

Our study demonstrates a clear improvement in cytogenotoxic damage in drug abusers supplemented with folic acid. The observed improvement could be attributed to the antioxidant capacity of folic acid, which counteracts the damage caused by the release of free radicals and direct DNA damage. Therefore, folic acid can be considered a potential intervention therapy to supplement vulnerable groups with drug abuse.

## Figures and Tables

**Table 1:** Demographic characteristics and drug consumption pattern.

Demographic Characteristics and Drug Consumption Pattern	Drug Abusers n = 44	Healthy Controls n = 44
Male	37	17
Female	7	27
Age (years)	29.54 ± 9.38	31.16 ± 9.36
Smoking Index	5.92 ± 6.61	---
SDU/day	4.85 ± 6.95	---
Marijuana grams/week	18.29 ± 27.65	---
Cocaine grams/week	1.32 ± 2.36	---
Methamphetamine grams/week	7.48 ± 9.59	---
Inhalants hours)/week	6.15 ± 14.76	---

The information on demographic variables and consumption patterns is expressed as mean ± standard deviation. Standard drink unit (SDU). Inhalants include thinner and polychloroprene yellow glue.

**Table 2:** Frequency of nuclear abnormalities in drug abusers and healthy controls.

Nuclear Abnormalities	Healthy Controls	Drug Abusers
NA/2000 cells	9.09 ± 3.79	28.45 ± 17.74 *p* < 0.001
MNs/2000 cells	0.45 ± 0.50	0.11 ± 0.32 *p* < 0.001
NBUDs/2000 cells	2.36 ± 1.90	3.20 ± 5.40 *p* < 0.005
BN/2000 cells	0.55 ± 0.66	2.66 ± 2.51 *p* < 0.0001
CC/2000 cells	2.36 ± 2.29	5.66 ± 5.48 *p* < 0.0005
KX/2000 cells	1.61 ± 2.13	14.07 ± 14.95 *p* < 0.0001
PN/2000 cells	0.55 ± 0.75	2.39 ± 2.85 *p* < 0.0005
KL/2000 cells	1.20 ± 1.31	0.36 ± 0.94 *p* < 0.0005

The data for nuclear abnormalities are expressed as mean ± standard deviation. The comparison between the healthy controls and the drug abusers was made with the Mann–Whitney *U* statistical test. Statistical significance was considered with a value of *p* < 0.05. Nuclear abnormalities (NAs), micronuclei (MNs), nuclear buds (NBUDs), binucleated cells (BNs), abnormally condensed chromatin (CC), karyorrhexis (KX), karyolysis (KL), and pyknotic nuclei (PNs).

**Table 3:** Frequency of nuclear abnormalities in baseline drug consumers (pre-treatment) vs. 15 and 30 days (post-treatment) with folic acid.

Nuclear Abnormalities	Baseline Drug Consumers (Pre-Treatment)	15 Days (Post-Treatment) with Folic Acid	30 Days (Post-Treatment) with Folic Acid
NA/2000 cells	28.45 ± 17.74	11.18 ± 7.42 *p* < 0.001	9.11 ± 10.9 *p* < 0.001
MNs/2000 cells	0.11 ± 0.32	0.05 ± 0.21 NS	0.05 ± 0.21 NS
NBUDs/2000 cells	3.20 ± 5.40	0.95 ± 1.72 *p* < 0.05	0.73 ± 1.04 *p* < 0.05
BN/2000 cells	2.66 ± 2.51	0.30 ± 0.79 *p* < 0.0001	0.75 ± 1.42 *p* < 0.0005
CC/2000 cells	5.66 ± 5.48	3.20 ± 2.80 *p* < 0.005	2.16 ± 2.54 *p* < 0.0005
KX/2000 cells	14.07 ± 14.95	5.82 ± 5.40 *p* < 0.0005	5.00 ± 7.77 *p* < 0.0005
PN/2000 cells	2.39 ± 2.85	0.80 ± 1.25 *p* < 0.005	0.23 ± 0.60 *p* < 0.0001
KL/2000 cells	0.36 ± 0.94	0.07 ± 0.25 NS	0.20 ± 1.36 NS

The data for nuclear abnormalities are expressed as mean ± standard deviation. The comparison between baseline drug abusers (pre-treatment) vs. 15 and 30 days (post-treatment) with folic acid was performed using ANOVA (Dunnett’s multiple comparisons). Statistical significance was considered with a value of *p* < 0.05. Nuclear abnormalities (NAs), micronuclei (MNs), nuclear buds (NBUDs), binucleated cells (BNs), abnormally condensed chromatin (CC), karyorrhexis (KX), karyolysis (KL), and pyknotic nuclei (PNs); no statistical significance (NS).

## Data Availability

All data generated or analyzed during this study are included in this article. Further inquiries can be directed to the corresponding author.
